# Expression of Emotions and Genes: Proinflammatory Gene Expression Rises With Spousal Distress

**DOI:** 10.1093/geroni/igab046.1148

**Published:** 2021-12-17

**Authors:** Stephanie Wilson, Steven Cole, M Shrout, Janice Kiecolt-Glaser

**Affiliations:** 1 Southern Methodist University, Dallas, Texas, United States; 2 UCLA School of Medicine, Los Angeles, California, United States; 3 The Ohio State University College Of Medicine, Columbus, Ohio, United States

## Abstract

Marital quality shares ties to inflammation-related conditions like cardiovascular disease and diabetes. Lab-based studies implicate hostility during marital conflict as a mechanism via inflammatory reactivity. However, developmental theories suggest that conflict declines with age. Spousal distress is an important but overlooked context for aging couples as networks shrink and assistance needs increase. To examine the effects of spousal distress on changes in proinflammatory gene expression, 38 adults ages 40-81 witnessed their spouse relive an upsetting personal memory aloud, rated their mood before and after, and provided blood samples at baseline and twice post-task. Those whose negative mood increased more in response to spousal disclosure showed larger elevations in proinflammatory gene expression 40 (p=.022) and 80 minutes (p<.0001) after the task. Effects were robust to race, gender, age, alcohol, smoking, and body mass index. These novel findings identify spousal distress as a key marital context that may escalate inflammation-related health risks.

